# Presynaptic Precursor Vesicles—Cargo, Biogenesis, and Kinesin-Based Transport across Species

**DOI:** 10.3390/cells12182248

**Published:** 2023-09-11

**Authors:** Astrid G. Petzoldt

**Affiliations:** Institute for Biology and Genetics, Freie Universität Berlin, Takustrasse 6, 14195 Berlin, Germany; astrid.petzoldt@fu-berlin.de

**Keywords:** presynaptic precursor vesicles, kinesins, Arl8/BORC complex, Rab2

## Abstract

The faithful formation and, consequently, function of a synapse requires continuous and tightly controlled delivery of synaptic material. At the presynapse, a variety of proteins with unequal molecular properties are indispensable to compose and control the molecular machinery concerting neurotransmitter release through synaptic vesicle fusion with the presynaptic membrane. As presynaptic proteins are produced mainly in the neuronal soma, they are obliged to traffic along microtubules through the axon to reach the consuming presynapse. This anterograde transport is performed by highly specialised and diverse presynaptic precursor vesicles, membranous organelles able to transport as different proteins such as synaptic vesicle membrane and membrane-associated proteins, cytosolic active zone proteins, ion-channels, and presynaptic membrane proteins, coordinating synaptic vesicle exo- and endocytosis. This review aims to summarise and categorise the diverse and numerous findings describing presynaptic precursor cargo, mode of trafficking, kinesin-based axonal transport and the molecular mechanisms of presynaptic precursor vesicles biogenesis in both vertebrate and invertebrate model systems.

## 1. Introduction

**Figure 1:**
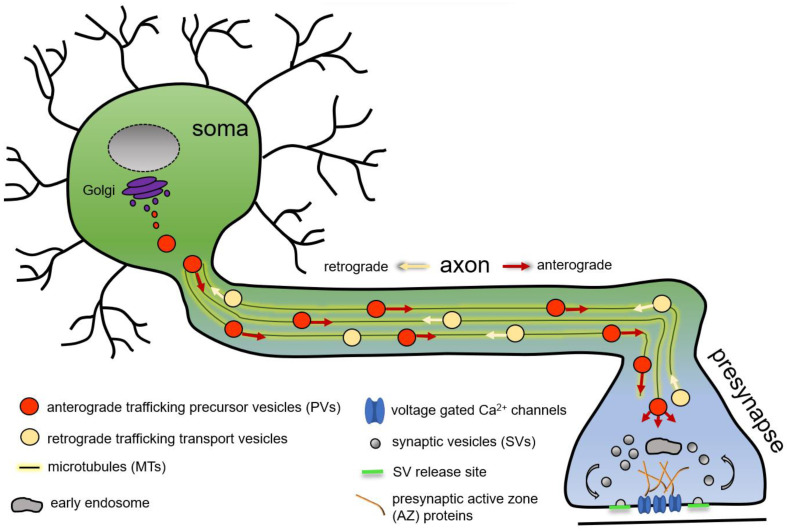
Schematic representation of microtubule-based axonal presynaptic precursor vesicle transport of presynaptic proteins from the neuronal soma to the consuming presynapse.

A neuron is a highly polarised cell with the cell body often distant from its synaptic terminals, where synapses are formed and maintained. Robust presynapse formation, maturation, and maintenance depend on the continuous delivery of presynaptic proteins from the neuronal soma, the predominant site of protein production. In the neuronal soma, newly synthetised presynaptic proteins are packaged into presynaptic transport vesicles or presynaptic precursor vesicles (PVs, this general term is used in this review to unitingly denominate all forms of presynaptic protein-transporting vesicles or organelles), exported from the soma to traffic anterogradely on polarised microtubules (MTs) along the axon to the consuming presynapse (Figure 1) [1,2,3,4,5,6,7,8,9].

Of note, although protein biosynthesis predominantly occurs in the neuronal soma, growing evidence is raised that pre- and postsynaptic local mRNA translation and protein synthesis significantly contribute to activity-dependent synapse plasticity mechanisms [10,11,12,13]. Nonetheless, despite the fact that mRNA transcripts are detected in axons and growth cones [14], presynaptic protein transcripts are not sufficiently enriched to solely account for the extensive protein demand of synaptic terminals [7,15]. This review focusses on presynaptic precursor vesicle cargos and morphology, the biogenesis of PVs in the neuronal soma, and PV trafficking along the axon, including kinesins implied in PV transport, and finally highlights a specific kinesin–precursor adapter/activator complex: the Arl8/BORC complex.

## 2. Presynaptic Precursor Vesicle Cargo, Morphology, and Transport Arrangements

### 2.1. Precursor Vesicle Cargo: Presynaptic Proteins

The presynapse is composed of a variety of structurally and functionally highly diverse proteins, including synaptic vesicle (SV) proteins with integral membrane and membrane-associated proteins, cytosolic active zone (AZ) proteins and release factors, ion-channels, adhesion molecules, and presynaptic membrane proteins coordinating vesicle exo- and endocytosis (Table 1) [2,3,8,16]. In numbers, it was estimated that an average presynapse in the vertebrate brain contains as many as approximately 300,000 molecules [17].

Scaffold proteins structure the presynaptic AZ, the site of SV fusion and neurotransmitter release, by defining SV release sites, clustering voltage-gated calcium channels (VGCCs or Cavs) in the centre of the AZ, and tethering SVs [16,18,19]. They comprise RIM-BP (Rab3-interacting molecule binding protein), the ELKS/CAST protein family (glutamic acid (E), lysine (K), leucine (L), and serine (S)-rich protein = ELKS; cytomatrix at the active zone (CAZ)-associated structural protein = CAST), including the ELKS/CAST homologue Bruchpilot (BRP) in *Drosophila*, the vertebrate specific proteins Piccolo and Bassoon, as well as Liprin-α/Syd-2 (LAR-interacting protein = Liprin; synapse defective 2 = Syd-2), Syd-1, and Spinophilin/Neurabin [16,18,19,20]. Release factors such as RIM (Rab3-interacting molecule) and (M)Unc13 (uncoordinated movement 13) concert the SV release, and (M)Unc18 (uncoordinated movement 18) and complexin regulate SNARE (soluble N-ethylmaleimide-sensitive-factor attachment receptor) complex activity [16,21,22,23,24]. Apart from these cytosolic proteins, several integral membrane proteins localise to different membranes and organelles at the presynapse. Fusion of SVs is triggered by local Ca^2+^ influx through voltage-gated Ca^2+^ channels upon arrival of an action potential and is executed by the SNARE-complex with its proteins residing in the SV (Synaptobrevin/Vamp) and presynaptic membrane (SNAP-25 = synaptosomal-associated protein, 25 kDa and Syntaxin) [23,24]. The SV membrane is crowded with integral membrane proteins [25] including Synaptotagmins as Ca^2+^ sensors, VGlut (vesicular glutamate transport protein), Synaptophysin, Synaptogyrin, SV2 (synaptic vesicle protein 2), and associated or peripheral membrane proteins, e.g., Rab3 (Ras-associated binding), CSP (cysteine string protein), and Synapsin [2,8,23,24]. Presynaptic transmembrane proteins further include synaptic adhesion molecules, e.g., N-cadherin and Neurexin, forming trans-synaptic connections to the postsynapse [2,3,16,23].

This plurality of presynaptic proteins, including integral or peripheral membrane and cytoplasmic proteins, is transported speedily by PVs to the synapse, as synapse formation itself is a rapid process and occurs within 30–60 min in vertebrate neurons after initial axodendritic contact formation [4,26], and in *C. elegans*, accumulation of presynaptic proteins in nascent synaptic specialisations occurs even within 5 min [27]. The fast delivery and synapse formation kinetic raise several conceptual questions: Are presynaptic proteins transported individually or in stoichiometric preassembled units? What is the cargo of a single PV? Are different PV types transporting different protein assortments, e.g., SV versus AZ proteins or membrane proteins versus cytosolic proteins? Would a difference in cargo load require different PV morphologies or are PVs uniform in size and shape? Is each individual PV transported singly by a specific kinesin or do PVs assemble into packets for axonal transport and delivery? None of these questions have been answered unambiguously to date; however, several concepts have been proposed, and a multiplicity of experimental data were collected over the last decades, spanning the different genetic model systems.

### 2.2. Presynaptic Precursor Vesicles: Ultrastructure and Cargo in Fixed Cells

**Figure 2:**
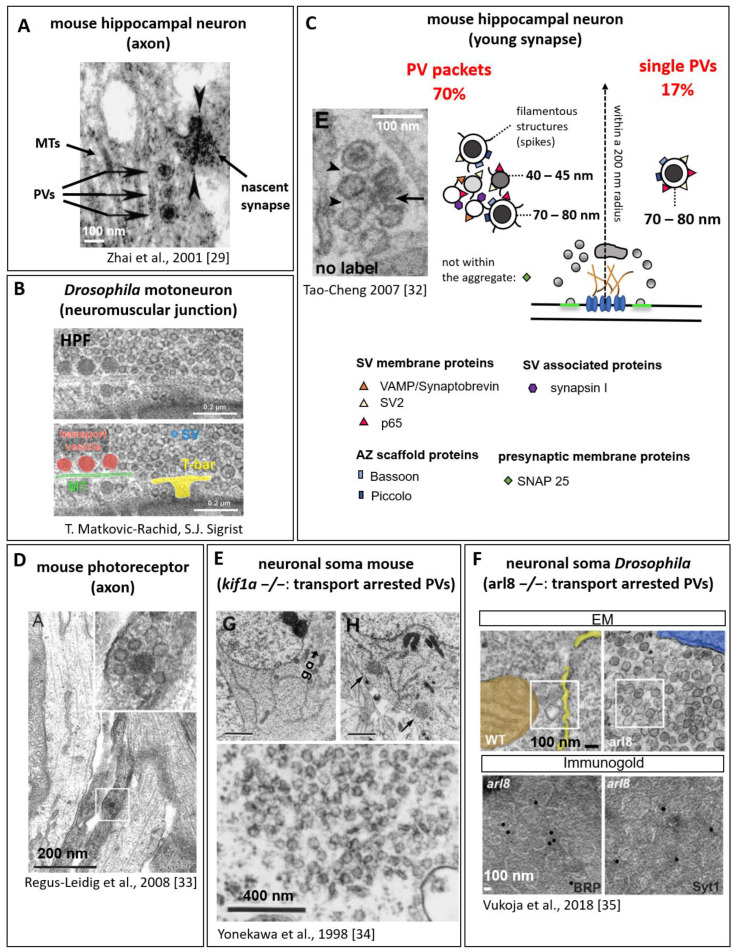
PV morphology and cargo. (**A**) EM micrograph from mouse hippocampal neurons and (**B**) from *Drosophila* neuromuscular junction (NMJ) showing microtubules (MTs) with associated ~80 nm dense cored vesicles in vicinity of a presynapse ((**A**) adapted from [29], (**B**) high-pressure freeze (HPF) micrographs from T. Matkovic-Rachid, S.J. Sigrist, unpublished). (**C**) EM micrograph and schematic summary of immuno-EM labelled PVs of young synapses from mouse neuronal cell cultures for various presynaptic proteins (adapted from [32]). (**D**) EM micrograph from an axon of mouse retinal photoreceptor neurons with PV units of multiple vesicles, (**E**) neuronal soma from mouse *kif1a*−/− with ectopically accumulating PVs, and (**F**) *Drosophila arl8−/−* mutants showing transport arrested PVs; lower panel with immunogold labelling for AZ protein Bruchpilot (BRP) and SV protein Synaptotagmin 1 (Syt1) ((**D**) adapted from [33]), (**E**) adapted from [34], (**F**) adapted from [35]). (Reprinted with permission from Ref. [29]. Copyright 2023 Elsevier. Reprinted with permission from Ref. [32]. Copyright 2023 Elsevier. Reprinted with permission from Ref. [33]. Copyright 2023 John Wiley and Sons. Reprinted with permission from Ref. [34]. Copyright 2023 Rockefeller University Press. Reprinted with permission from Ref. [35]. Copyright 2023 Elsevier.)

Early studies in 1989 visualised by electron microscopy (EM) synapses of the mouse CNS (central nervous system) and described “granulated” vesicles of 60–80 nm size and “empty” vesicles with varying size, morphology, and electron density localising preferentially near the presynaptic membranes, suggested to deliver presynaptic material [28]. Of note, the granulated vesicles are thought to be distinct from the neuropeptide containing “classical” dense core vesicles (DCVs) [28,29]. Subsequent EM studies in cultured hippocampal neurons observed 80 nm dense cored vesicles stringed on MTs in the vicinity of nascent synapses (Figure 2A) [29], and a similar PV scenario is observable at neuromuscular synapses in *Drosophila* (Figure 2B). First PV cargos were identified: the AZ scaffold protein Piccolo was detected by immunogold labelling on the outside of 80 nm granulated (dense cored) vesicles, hence the denomination of the characterised vesicles as Piccolo transport vesicles (PTVs) [29]. This EM study further suggests that the presynaptic cytoplasmic proteins localise to the outside of the precursor vesicle and are not transported in the vesicular lumen. The mechanisms by which the cargo proteins are hooked on the membrane are currently not known. Immunoisolated Piccolo-PVs were biochemically positive for other presynaptic proteins, including the AZ scaffold protein Bassoon, the presynaptic membrane SNARE proteins Syntaxin and SNAP-25, and the cell adhesion molecule N-cadherin (see Table 1), while SV proteins (Synaptobrevin/Vamp, Synaptotagmin) were not detected [29]. Immunoisolated Bassoon-PVs were positive for RIM1 and ELKS/CAST, but not for (M)Unc13-1 or Synaptophysin [30]. Using the same technique, electron micrographs of immunoisolated PVs were additionally positive for the cytoplasmic SV release controlling proteins (M)Unc18, (M)Unc13, and RIM, the peripheral SV membrane protein Rab3, and the α1 and β1 subunits of the N-type Ca^2+^ channel [31]. These findings were confirmed by confocal immunofluorescence microscopy showing a partial overlap of Piccolo-positive puncta with Rab3, (M)Unc18, and RIM, and immunoisolated Piccolo-PV were positive for Bassoon, RIM, and (M)Unc18, as detected by immuno-EM analysis [31].

Appart from the detection of single PVs and the identification of their cargo, several observations suggest that axonal PV transport occurs in packets or aggregates of differently shaped PVs. In hippocampal neurons, using correlating fluorescent light microscopy and retrospective EM, “transport packets” were observed, composed of several separate membranous organelles including dense cored vesicles (70 ± 10 nm diameter), pleiomorphic clear cored vesicles of 42 ± 15 nm × 22 ± 13 nm size, and tubulovesicular membrane-structures [4]. These packets transported Synaptobrevin/Vamp, voltage-dependent calcium channel subunits, SV proteins (SV2, Synapsin 1), and an endocytosis protein (Amphiphysin 1) [4]. A subsequent study immunolabelling PVs in neuronal cell cultures identified, by EM, aggregates of one to two 70 nm sized dense cored vesicles surrounding five to six clear cored and smaller vesicles, which occure with a 70% frequency at young presynapses within a 200 nm radius of the presynaptic centre [32]. Within the PV aggregates, SV membrane proteins Synaptobrevin/Vamp, SV2, Synaptotagmin/p65, and the SV-associated protein Synapsin 1 localised to the clear cored 45 nm vesicles, but also to the larger dense cored vesicles, while the presynaptic membrane protein SNAP-25 was not detected in the aggregates (Figure 2C) [32]. Filamentous structures or spikes seemed to connect the single vesicles. Single 70 nm sizeddense cored vesicles positive for Bassoon and Piccolo were observed at a lower frequency of 17%,, with a decreasing frequency from young to old synapses (Figure 2C) [32]. Interestingly, when quantifying protein labelling on single 70 nm sized dense cored PVs, Bassoon (5%) and Piccolo (17%) were, compared to SV2 (88%) and Synaptotagmin/p65 (56%), less frequently present, indicating that individual PVs are unlikely to account for the majority of presynaptic active zone protein transport [32].

Studies in mouse retinal photoreceptor ribbon synapses using STED (stimulated emission depletion) microscopy and immuno-EM also identified assembled PV transport units with a total diameter of 130 nm composed of multiple vesicles positive for Bassoon, Piccolo, Ribeye, and RIM, but not for (M)Unc13 and ELKS/CAST (Figure 2D) [33]. It is assumed, that these units represent an early stage in the formation of synaptic ribbons. The diverse and varying observations suggest that PVs can probably traffic as single entities and, possibly more frequently, as macromolecular complexes of preassembled presynaptic proteins in PV packets composed of several different PV types to supply the presynapse with the required components (summarised also in [2,5,8]).

A possibility to visualise PVs in higher numbers compared to occasional axonal transport events arises when the transporting kinesin or kinesin adaptor (for details, see also later paragraphs) are depleted, inducing an accumulation of “transport arrested” PVs in the neuronal soma. In knockout mice for the anterograde kinesin-3 motor KIF1A, small, clear cored vesicles accumulated in clusters in the neuronal soma (Figure 2E) [34]. Similarly, knockout of the kinesin adaptor Arl8 in *Drosophila* leads to an accumulation of PV clusters in the neuronal soma [35]. Electron micrographs here reveal uniform-shaped vesicles of ~70 nm diameter with all shades from clear to dense cored vesicles. Immunogold labelling confirmed both AZ proteins (BRP) and SV proteins (Synaptotagmin 1) as PV cargo (Figure 2F) [35]. Future comparative analysis between species might help to unify the different observations into a comprehensive model.

### 2.3. Live Imaging of Presynaptic Precursor Vesicle Trafficking

Additionally tothe immobile snapshots of PVs at the presynapse, in the axon, or the soma, live imaging analysis provides valuable insights into the cargo load of PVs, easily identified by co-trafficking analysis of fluorescently tagged proteins. The first live imaging experiments overexpressing GFP-tagged Bassoon already revealed that single PVs carry 50% of Bassoon, Piccolo, and RIM protein incorporated in a mature synapse, suggesting that two to three PVs deliver sufficient protein for a single synapse formation event [31]. However, a high observed variance suggests that AZ assembly is not a classical “quantal” process, albeit based on presynaptic material delivery through integer numbers of PVs [31]. In later studies, co-trafficking of Bassoon and ELKS/CAST, but not (M)Unc13, was observed [36]. Neurexin and Ca^2+^ channels, as shown by a study in hippocampal neurons, described epitope-tagged Neurexin co-trafficking with RIM1α and N-type Ca^2+^ channels, but not Bassoon [37]. Additionally, fluorescently tagged Neurexin showed a 60–75% co-labelling with the SV proteins Synaptophysin, Synapsin, and Synaptotagmin [38]. In *Drosophila* motoneurons, the AZ proteins BRP and RIM-BP are co-transported, as detected by intravital axonal imaging [39], and in *C. elegans*, in vivo analysis described co-transport of two SV proteins, Rab3 and Synaptogyrin [40]. As a summarising model integrating EM immunolabelling and life imaging data, the existence of two or three PV types with distinct cargos was suggested: (i) large, dense cored, 70–80 nm sized AZ-protein-transporting PVs, denominated PTVs or AZ precursor vesicles, charged with ELKS/CAST, Bassoon, Piccolo; (ii) small, clear cored, 30–40 nm sized SV-protein-transporting PVs, denominated SVP (synaptic vesicle precursors) transporting Synaptobrevin/Vamp, SV2, Synaptotagmin/p65, Synapsin, possibly also voltage-gated Ca^2+^ channels, RIM, and Neurexin; and (iii) only (M)Unc13 specific PVs [2,8,9,20,41].

Recent studies investigated co-trafficking of both SV and AZ proteins. An in vivo study in both vertebrate and invertebrate systems revealed axonal co-trafficking of presynaptic AZ and SV proteins, with Bassoon and VGlut (vesicular glutamate transport protein) in hippocampal neurons and the ELKS/CAST homologue Bruchpilot and Synaptotagmin 1 in *Drosophila* motoneurons [35]. Also, in *C. elegans*, dual-colour live imaging showed co-transport of RIM/Unc10 with Rab3 (95% of all RIM/Unc10-positive PVs) and Synaptogyrin, and Liprin-α/Syd-2 and Rab3 co-trafficking [42]. The observed AZ/SV “co-trafficking” could either result from single PVs carrying both cargos, in agreement with the immuno-EM analysis showing both AZ and SV proteins on a single PV (Figure 2C) [32] or represent the multivesicular “transport packets” of different PVs with either SV or AZ protein cargo (see Figure 2C) [32,41]. Alternatively, it has been suggested that additional maturation steps during the axonal transport prior to presynaptic protein incorporation possibly constitute SV and AZ containing PVs [2]. However, recent live imaging of AZ (ELKS-1, Liprin-α/Syd-2, RIM/Unc10) and SV proteins (Rab3) in *C. elegans* revealed different trafficking kinetics for the two protein groups in the area behind a growth cone [27]. AZ proteins showed a slow and infrequent transport of large puncta, compared to SV proteins, and did not feed directly into forming synapses, but into a diffuse pool, suggesting that AZ and SV proteins use distinct PVs with distinct motor proteins during axonal transport.

Finally, it should be noted that next to the delivery of newly synthesised proteins from the soma, local shuffling of presynaptic material occurs, both between individual AZs, and between AZ and extrasynaptic, cytosolic reservoir pools [7,43,44,45,46]. This local rearrangement of presynaptic material does not require an “on locus” delivery of a precise number of PVs to form an individual synapse. Possibly, AZ proteins tend to be transported in quantal large PVs to these local reservoir pools, while SV proteins traffic on smaller precursor vesicles directly to the nascent or growing presynapse [7]. However, presynaptic cargo needs to be delivered to either the local reservoir pool or directly to the consuming presynapse, and distinct, but not exclusive, modes of protein supply might be utilised during synaptogenesis, possibly dependent on the physiological properties of a synapse (tonic versus phasic synapses) and/or the developmental status of the synapse (initial synapse seeding versus nascent synapse growth versus mature synapse maintenance).

## 3. Presynaptic Precursor Vesicle Biogenesis

**Figure 3:**
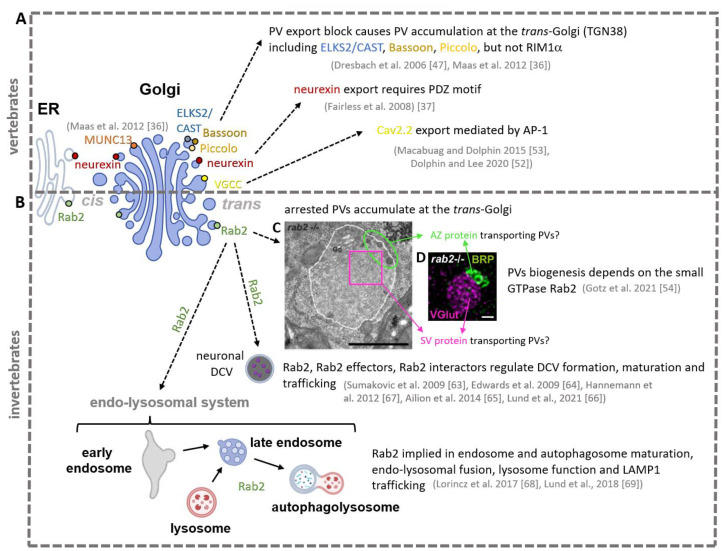
Biogenesis of presynaptic precursor vesicles at the *trans*-Golgi in (**A**) vertebrate and (**B**) invertebrate systems. Summary of the collective evidence in vertebrates and invertebrates of presynaptic precursor vesicles assembly at the *trans*-Golgi from [36,37,47,52,53,54,55,56,57,58,59,60,61]. Small GTPase such as Rab2 might play a central role in vesicular biogenesis at the *trans*-Golgi by orchestrating multiple molecular export routes, including DCV maturation, endo-lysosomal formation and maturation, and PV biogenesis (drawings created with BioRender.com). Scalebar: (**C**) EM micrograph 500 nm, (**D**) confocal immunofluorescence staining 0.5 µm. For copyright notice, see paragraph at the end of the article. (Reprinted with permission from Refs. [36,37,47,52,53,54,55,56,57,58,59,60,61]. Copyright 2023 Journal of Cell Biology).

Assembly of PVs occurs in the neuronal soma, and although the underlying molecular pathways controlling formation of the membranous transport organelles have been intensely studied, the current biogenesis model is still fragmentary. A set of early studies in cultured mouse neurons provided evidence that PVs originate from the *trans*-Golgi (Figure 3) [31,36,47]. Proteins are processed in the Golgi and subsequently sorted at the *trans*-Golgi on different carriers or transport vesicles, and finally exported towards their specific destination, e.g., plasma membrane or the endo/lysosomal system [48,49,50]. It has been estimated that in cells of cultured cell lines, roughly 4000 proteins per second are leaving the Golgi [51]. In cultured hippocampal neurons, endogenous Bassoon and Piccolo colocalise with the *trans*-Golgi marker (TGN38) upon controlled cooling-induced attenuation of Golgi-export, causing an increase of the Golgi-associated Bassoon protein fraction and a decrease of Bassoon level in axons, while drug-induced Golgi disruption dispersed Bassoon in the cytoplasm [47]. Later studies utilising the same experimental paradigm to attenuate Golgi-export showed a diverse Golgi localisation pattern for different presynaptic proteins: the AZ protein ELKS2/CAST localised similar to Piccolo and Bassoon at the *trans*-Golgi, while (M)Unc13 localised to the *cis*-Golgi and RIM1α was not detected at the Golgi [36]. Neurexin was shown to be enriched in the endoplasmatic reticulum (ER)/Golgi, and ER/Golgi export depends on the PDZ-binding motif (postsynaptic density protein (PSD95), *Drosophila* disc large tumour suppressor (Dlg1), and zonula occludens-1 protein (zo-1)) of the Neurexin C-terminus [37]. Furthermore, voltage-gated Ca^2+^ channels are thought to be packaged on PVs at the *trans*-Golgi and transported towards the presynapse [52], and for N-type voltage-gated calcium (CaV2.2) channels, trafficking from the *trans*-Golgi is mediated by the Adaptor protein complex-1 protein (AP-1) [53].

A recent study on *Drosophila* identified the small, Golgi-related GTPase Rab2 (Unc108 in *C. elegans*) as a crucial regulator of the early steps of PV biogenesis at the *trans*-Golgi [54]. Presynaptic precursors accumulate at the *trans*-Golgi upon Rab2 knockdown and synaptic terminals show reduced presynaptic protein level. Interestingly, ultrastructural EM analysis of these maturation arrested PVs in the soma showed a large subfraction of elongated, sometimes tubular clear cored vesicles of 40–60 nm size and a small population of circular, large, dense cored vesicles of roughly 80 nm size (Figure 3) [54]). Immunofluorescent co-labelling revealed an interesting cargo segregation within the ectopic PV clusters: a large PV fraction was co-positive for synaptic vesicle proteins (VGlut, Sytaptotagmin 1), the endocytic machinery (Dap160/intersectin, dynamin-associated protein 160 kDa), and the lysosomal marker Lamp1 opposed to small PV fraction co-positive for the presynaptic AZ proteins BRP, RIM-BP, and (D)Unc13A (*Drosophila* Unc13), homologue of the vertebrate (M)Unc13. Thus, possibly already in this early step of PV biogenesis during protein export from the *trans*-Golgi, two types of PV are differentiated: 40–60 nm sized clear cored synaptic vesicle transporting PVs and 80 nm sized dense cored AZ-protein-transporting PVs (Figure 3) [20,31,32,41]. However, without ultrastructural labelling analysis, this remains an assumption.

Small GTPases including Rab2 recruit in their activated GTP-bound state effector proteins to dedicated membranes to concert vesicle fusion and/or fission events [62,63], and might hence play a central role in the organisation of vesicle formation and trafficking at the *trans*-Golgi [64].

Early studies described Rab2 as a Golgi-resident to act in anterograde and retrograde ER-Golgi trafficking [64,65,66]. However, several lines of evidence expand Rab2 function to vesicular biogenesis pathways at the *trans*-Golgi, including PV biogenesis but also the formation of neuronal DCVs. DCVs have a typical size of roughly 43 nm diameter with a dense core undergoing a complex maturation sequence to form mature neuropeptide transporting organelles [67,68,69]. In the *C. elegans* system, Rab2/Unc108 is involved in neuronal DCV biogenesis at the *trans*-Golgi, as DCV morphology (size and shape) and PI(3)P-dependent cargo sorting during DCV maturation is aberrant in *rab2−/−* mutants [55,56]. Importantly, Rab2 effectors or interactors, such as RIC-19 (resistance to inhibitors of cholinesterase-19), Rund-1 (RUN domain-containing protein 1), CCCP-1/Golgin104 (conserved coiled-coil protein 1), and TBC-8 (Tre2/Bub2/Cdc16 domain containing protein-8), are implied in DCV maturation at the *trans*-Golgi [55,57,58,59]. Also, axonal transport of DCVs and lysosomes in *Drosophila* neurons depends on Rab2 [58]. Finally, Rab2 was shown to be required for autophagosome and endosome maturation as well as lysosome function in non-neuronal *Drosophila* tissue [60], and endo-lysosomal fusion and the delivery of the lysosomal protein Lamp1 to late endosomes [61]. Interestingly, PVs transport was shown to depend on the lysosomal kinesin adaptor Arl8, and PV membranes contain lysosomal membranes proteins such as Lamp1 or Spinster (for details, see last paragraph) [35,70]. As lysosomal proteins, e.g., Lamp1, are exported from the *trans*-Golgi on lysosomal membrane protein (LMP) carriers, a process mediated by Rab2 [61] and, Rab2 is implied in lysosome formation, while PVs share a lysosome-like membrane identity, e.g., contain Lamp1 [35], Rab2 might at the *trans*-Golgi organise PV biogenesis using components of the other Golgi export pathways, or, as part of the PV maturation process, mediate fusion events with endo-lysosomal organelles.

In summary, these findings place the small GTPase Rab2 at a potent cross-section of three organelle biogenesis pathways, intersecting PV, DCV, and endo-lysosome biogenesis and trafficking, potentially acting here as a signpost or decision-maker concerting an interplay of *trans*-Golgi export routes, possibly using components of the hitherto independently described molecular pathways to orchestrate PV biogenesis (Figure 3).

## 4. Axonal Microtubule Architecture and Regulation of Kinesin Activity Concert Presynaptic Precursor Vesicle Trafficking

Mature PVs exit the neuronal soma and are subsequently transported anterogradely on MTs along the axon towards the consuming presynaptic site (Figure 1). Quantitative transport analysis of PVs in vertebrate neurons estimates that, per day, 1.5–9 million SV-protein-positive PVs traffic along the axon and 17–35 of these precursors are generated per second in the neuronal soma [1]. The molecular machinery regulating the axonal transport has been intensely investigated. Neuronal MTs typically consist of 13 protofilaments, composed of α- and β-tubulin heterodimers, which assemble head-to-tail, creating a polar ~25 nm wide tube with a minus-end (α-tubulin) and a plus-end (β-tubulin). MT filaments grow and shrink continuously due to association or dissociation of heterodimers, with the plus-end being more dynamic and the minus-end being more stable [1,71,72]. Directional transport along MTs into axons or dendrites depends, among others, on the distinct, local MT architecture. A prominent example here is the selective cargo transport into dendrites based on the presence of minus-end-outward pointing MTs [73,74]. Next to MT polarisation, posttranslational modifications also influence MT architecture and, thus, directional transport, e.g., in dendrites where acetylated MTs are stable and mostly pointing with the minus-end out, while the dynamic MTs are tyrosinated with a plus-end-out orientation [75].

Axons typically contain bundles of 10–100 MTs with a plus-end-out direction, as observed in different model organisms [72]. MT-based transport is driven by two types of cytoplasmic motor proteins: kinesins moving to MT plus-ends for anterograde organelle transport, and dynamins moving to MT minus-ends for retrograde transport [76,77,78]. Kinesins form a large protein superfamily, with ~45 different kinesins expressed in mammals. Axonal transport is composed of two kinetically distinct components, the “fast” transport with velocities above 0.5 µm/sec (typically 1–3 µm/s) for organelles, such as mitochondria or PVs, in contrast to transport of soluble or cytoskeletal proteins with a velocity of <0.1 µm/sec representing the “slow” trafficking component [79]. Kinesins have selective preferences for different types of MTs and their associated proteins, e.g., Kinesin-1 (KIF5) preferentially transports along acetylated MTs [80], while Kinesin-3 (KIF1A) favours tyrosinated MTs [75,81]. Efficient long-range transport of presynaptic cargo along the axon is based on comparably long and stable MTs, while at the synaptic terminal, shorter and dynamic MTs with plus-tips allow for slower transport and cargo arrest, promoting presynaptic cargo incorporation into the AZs [1].

Members of the kinesin-1 and -3 family are the major MT motors implied in presynaptic cargo transport. Motor domains of both kinesin-1 and -3 family members are localised at the N-terminus [82,83]. Kinesin-1 family members (KIF5 in vertebrates and their homologues kinesin heavy chain (KHC) or uncoordinated movement (Unc)116 in invertebrates, see Table 2)) are homodimers and associate with two kinesin light chains (KLC), linking them to the cargo. However, the KHC itself can also bind to a cargo via specific adaptor proteins, as shown in case of Milton for mitochondrial transport [82,83]. Kinesin-3 (KIF1A in vertebrates and their homologues in invertebrates Unc104 or Imac = immaculate connections) family members can act as a monomer or dimer and bind either directly to the cargo or via adaptor proteins [82,83]. Retrograde transport depends on a single motor protein, the cytoplasmic dynein. Overall organelle or vesicle motility along an axon is determined by a “tug-of-war” scenario between anterograde- and retrograde-directed motors, kinesins and dynamins, respectively, as both motors can bind simultaneously to a cargo organelle [84,85].

Each kinesin cargo, such as mitochondria, lysosomes, or PVs, requires a combinatorial read-out of several molecular signals determining the individual cargo binding to the appropriate motor protein based on specific adaptor proteins in combination with MT-associated proteins (MAPs). This allows the fine-tuning of the kinesin activity, and motility to ensure accurate cargo load for cell- and synapse-specific cargo delivery. Kinesin-1 (KIF5A/B/C) and kinesin-3 (KIF1A) activation and termination have been extensively studied [1,83,118]. Kinesin activation is of particular importance for cargo exit from the neuronal soma, entry into the axon and anterograde transport to the release sites and the possibility to by reinitialise pausing cargo in the axon [1]. Both kinesins are, as free cytosolic components, inactive due to autoinhibition. A conformational change upon cargo binding permits MT association, initiated by electrostatic interaction between the kinesin motor domain (+) and MTs (−), stabilised by ATP hydrolysis [119,120]. Additionally, kinesin activity depends on several interacting proteins, motor subunits, and accessory proteins. As an example, Kinesin-3 can be inactivated by kinesin-specific binding of Kinesin-binding protein (KBP) to its motor domain, thus inhibiting MT binding [121]. Prominent examples of the regulative effect of MAP proteins are the axonal proteins Tau and MAP7, either inhibiting the binding of both Kinesin-1 and -3 to the MTs, in case of Tau, or for MAP7, inhibiting Kinesin-3 MT-binding, but promoting MT-binding for Kinesin-1 [122,123].

## 5. Kinesins and Their Presynaptic Precursor Vesicle Cargos

Specificity of kinesin-dependent axonal PV transport requires a balanced regulatory machinery composed of kinesin-adaptor/regulatory protein complexes. During the last decades, a wealth of data was collected and numerous studies in different model systems using a wide set of molecular markers have expanded our understanding of the kinesin-based PV transport. Although it will not be possible to summarise all findings (for detailed reviews, see [1,2,5,82,118,124], the core ideas and experiments shall be summarised here. The focus lies on members of the kinesin-1 and -3 family as major PV transporting kinesins, subdivided into vertebrate and invertebrate studies (see also Table 2).

### 5.1. Kinesin-3 Family

#### 5.1.1. Vertebrates (KIF1A/B)

Early studies in the vertebrate system provide evidence that KIF1A, the fastest anterograde motor protein, is associated with SV protein containing organelles positive for Synaptotagmin, Synaptophysin, and Rab3A, but not SV2 or the presynaptic membrane proteins Syntaxin 1 and SNAP-25 (see Table 1 and Table 2) [87]. However, later studies could show that both SV proteins Synaptophysin and SV2 are KIF1A cargos, as in *kif1a−/−* mutant mice, which are lethal after birth, transport of both proteins was disrupted and, consequently, SV proteins accumulated in the cell body and were absent from the synapse [34]. Also, KIF1B was shown to associate with the SV proteins Synaptotagmin, Synaptophysin, and SV2, but not with SNAP-25 and Syntaxin, as inferred from GST-pulldown and EM immunogold-labelling analysis [91]. *Kif1b−/−* mice displayed several neuronal defects and a strong reduction of Synaptotagmin and SV2 at the nerve terminal, while SNAP-25 and Syntaxin were consistently not altered [91]. Analysis of hippocampal neurons in microfluid system-based cell cultures allowed for live imaging analysis of single axonal organelles and confirmed that KIF1A transports Synaptophysin-positive vesicles, Synaptobrevin/Vamp, and SV2 transporting carriers [95]. Delivery of Synaptophysin-positive PVs depends on KIF1A, as a point mutation weakening kinesin-MT binding disrupted PV delivery to the presynapse, as shown by TIRF (total internal reflection fluorescence) imaging of hippocampal neuron cell cultures [96]. Notably, a direct interaction of KIF1A with Liprin-α (isolated from rat brains) was detected in coimmunoprecipitation assays, and additionally a Liprin-α association with RIM, possibly hooking RIM via Liprin-α to KIF1A [92].

#### 5.1.2. Invertebrates (Unc104, Imac)

Similar to vertebrates, in *C. elegans*, kinesin-3 (Unc104) is thought to act as major kinesin transporting PVs with an SV protein cargo [5,82,118,125]. Upon Unc104 depletion, few SVs are observed at the synapse, and synapse number and size are reduced, while SVs accumulate in the cell body [100]. KIF1A (Unc104) and another Kinesin-3, KIF1Bβ, were shown to transport Rab3-positive PVs in a DENN/MADD (Rab3-GEF, guanosine exchange factor)-dependent manner [111]. Anterograde transport of the SV proteins Synaptotagmin and Cysteine string protein (CSP) was disrupted in *unc104* mutants [126], and transport of Synaptobrevin-positive PVs was shown to be driven by Kinesin-3 (Unc104) and depended on Liprin-α/Syd-2 [112]. In *C. elegans*, depleting Unc104 using the canonical unc-104^D1497N^ allele with a mutation of the PH (pleckstrin homology) domain that diminishes cargo binding and causes protein degradation led to the accumulation of the SV protein Synaptogyrin in the neuronal soma, while its axonal transport was abolished [40]. Rab3 trafficking was also disrupted in a different *unc104* mutant, where Rab3 accumulated in the neuronal soma and proximal axon and, concomitantly, overexpression of Unc104 or gain-of-function mutations showed enhanced Rab3 transport with a decrease of the somatic and an increase of distal axonal and synaptic Rab3 [113]. A recent study showed that axonal transport of Neurexin is also mediated by Unc104 [114].

Other experiments shed light on how Unc104 can bind membranes of SV transporting PVs. Unc104 contains a C-terminal PH domain that specifically binds phosphatidylinositol-4,5-bisphosphate (PI(4,5)P2) [127]. A subsequent study in *C. elegans* revealed that this binding is required for anterograde Synaptotagmin transport, as deletion or point mutation of this PH domain abolishing PI(4,5)P2 binding resulted in a failure of Synaptotagmin transport and a consistent accumulation in the neuronal soma [109]. The Arl8/SKIP/BORC complex regulation of Kinesin-3 activity and cargo load is summarised separately and in detail in the last paragraph.

Supporting the Unc104 loss of function analysis, overactivation of KIF1A/Unc104 by disruption of the intramolecular autoinhibition of KIF1A/Unc104 causes an increase of SVs in dendrites [115]. Hyperactivation of Unc104 through the introduction of mutations causing the human disease hereditary spastic paraplegia disease (SPG) led to the accumulation of the SV protein Synaptobrevin in the neuronal tip [116]. These data suggest that Unc104 is the major anterograde motor for SV-protein-transporting PVs, raising the question as to whether AZ protein transport would also depend on Unc104.

In a recent *C. elegans* study, *unc104*−/− mutants showed no defect in axonal ELKS-1 distribution; however, RIM/Unc10 showed a mild accumulation in the proximal axon, similar to the SV protein gradient observed [27], suggesting that at least some AZ proteins are transported independently of Unc104. In *Drosophila*, Unc104 is required for both AZ and SV protein delivery to the synapse. In *unc104−/−* mutants, synaptic terminals (neuromuscular junctions = NMJs) were atrophic, boutons were not forming and devoid of SVs, and presynapses reduced in number in both embryonic and larval neurons [103,104]. Upon Unc104 depletion, protein levels of the SV proteins Synaptotagmin and VGlut and the AZ protein BRP were strongly reduced at the synaptic terminal and in the axon but accumulating in the neuronal soma [103,104,106]. Additionally, studies in hypomorph *unc104* mutants provide evidence that the presynaptic AZ protein Liprin-α and the VGCC Cacophony (Cac) are also transported by Unc104 [106], suggesting that Unc104 could also be implied in the trafficking of PVs with an AZ protein or VGCC cargo.

Notably, a recent study suggests an additional signalling role of Unc104 possibly causing the above summarised phenotypes independent of an Unc104 transport function. The “Wallenda (Wnd)/DLK MAP kinase axonal damage signalling pathway” is activated upon Unc104 depletion, and this activation was proposed to account for the observed reduction of presynaptic proteins at the synaptic terminal due to reduced expression levels of AZ and SV proteins [117].

### 5.2. Kinesin-1 Family

#### 5.2.1. Vertebrates (KIF5)

KIF5 is the primary kinesin motor for transport of mitochondria and lysosomes [128,129], with KIF5B-deficient mice being embryonic lethal and displaying severe defects in mitochondrial and lysosomal localisation [130]. Nevertheless, there are several lines of evidence for an axonal PV transport function for kinesin-1 family members. KIF5B was shown to transport the presynaptic membrane protein Syntaxin 1 linked to KIF5B via the Syntabulin linker [86]. In a yeast-2-hybrid screen, SNAP-25 associates with KIF5B [89]. In HeLa cells, KIF5B also interacts with SNAP-25 and affects trafficking of SNAP-25 carrying vesicles [90], and, SNAP-25 was identified in a Syntabulin GST pulldown [86]. Later studies revealed that the AZ protein Bassoon is also transported by the Syntabulin/KIF5B complex [88]. Additionally, KIF5C binds Syntaxin 1 and (M)Unc18 via the adaptor protein FEZ (Fasciculation and elongation protein zeta 1, Unc76), a process regulated by FEZ phosphorylation [93]. Also, Bassoon, Piccolo, and (M)Unc13 are likely transported by Kinesin-1/FEZ as they co-accumulate in FEZ depleted mouse brains [94]. Finally, a comprehensive study identifying SV associated proteins by mass spectrometry on SVs isolated from rat brains detected only KIF5A/B, but not KIF1A [25], suggesting that kinesin-1 could also be implied in SV-protein-containing PV transport.

#### 5.2.2. Invertebrates (KHC, Unc116)

As in vertebrates, *Drosophila* kinesin-1 (Kinesin heavy chain = KHC) is crucial for anterograde transport of mitochondria [128,131]. Additionally, KHC is proposed to transport presynaptic proteins as mutations cause a severe reduction of synapses number and neuromuscular terminals are disrupted [99]. Hypomorph mutations (surviving until the third larval stage) as well as null mutants become paralyzed, a phenotype which increases towards the distal segments, causing the prominent upward flipping of the tail [99,132]. The proximal–distal phenotype gradient is typical for axonal transport defects as longer axons connecting to the distal terminals are more severely affected by reduced presynaptic material transport. In the case of KHC reduced voltage-gated sodium channel transport and, consequently, activity, causethe impaired action potential propagation [97,98,132]. In contrast to above described Kinesin-3 (Unc104), which does not show tail flipping, impairment of KHC causes occasional giant axonal swellings with a 10-fold increase of axon diameter containing numerous membranous vesicles, including multivesicular bodies, smaller, clear, and dense cored vesicles, and large dense cored lysosome-related vacuoles [99]. The SV proteins Synaptotagmin and CSP, the presynaptic membrane protein Syntaxin, and the extracellular presynaptic membrane protein Fasciclin-II (Fas-II) accumulate in these axonal swellings of the hypomorph *khc* mutants [99]. However, SV numbers at the terminal were not affected according to EM analysis [98]. It has been suggested that the observed giant axon swellings of *khc* mutants [99] are causing an axonal “traffic jam”, responsible for the impaired presynaptic protein transport. Interestingly, recent studies have revealed that KHC next to its cargo transporting function performs “MT sliding”, where “cargo” MTs are transported via Kinesin-1 along “track” MTs, a process required for axonal outgrowth [110,133,134]. In an elegant study, the C-terminal MT binding of KHC required for MT sliding was mutated, while the cargo binding function of KHC remained intact (*khc^mutA^*). Interestingly, the axonal traffic jam was now removed and, at least, SV proteins (Synaptotagmin and CSP) did not accumulate in axons anymore, implying a restoration of SV protein trafficking [110]. Notably, a recent study suggested that KHC/Unc116, at least in worms, is not required for transport of the AZ proteins ELKS-1 and Liprin-α/Syd-2, as in *unc116−/−* mutans both proteins still localise along the entire axon and are not accumulating in the proximal axon [27]. Even in double mutants for both kinesins (Kinesin-1/Unc116 and Kinesin-3/Unc104), ELKS-1 still trafficked into the entire axon [27], suggesting that neither kinesin is required for ELKS-1 transport.

In *Drosophila*, alike to the findings in vertebrates, the kinesin adaptor protein FEZ/Unc76 was shown to bind the C-terminus of KHC, and loss of FEZ/Unc76 caused defects in axonal transport of SV proteins, e.g., Synaptotagmin 1 accumulated in axons [101]. Additionally, FEZ/Unc76 depletion disrupted PV transport as Synaptotagmin 1- and CSP-positive PVs accumulated in the axon and were absent from the neuromuscular terminal [102]. Coherently, in *C. elegans*, loss of FEZ/Unc76 function caused axonal transport defects of Syntaxin 1 as it accumulated in axons and neuronal cell bodies. [93].

KHC interacts with specific cargo binding adaptors, such as the JNK scaffolding adaptor JIP-1 (JNK-interacting protein 1), regulating c-Jun N-terminal kinase (JNK) activity, as shown in vertebrates [135,136]. Functionally impeding or enhancing the JIP-1 *Drosophila* homologue APLIP-1 (APPL-interacting protein 1) phenocopies the *khc* mutant phenotype, including tail flipping, axonal swelling, and accumulation of the SV proteins Synaptotagmin and Synaptobrevin [107,108] and AZ proteins BRP and RIM-BP [39] in the axon. However, these findings could still be explained by the “traffic jam” hypothesis instead of a direct KHC transport function, and in vertebrates, JIP proteins are not implied in SV protein trafficking as *jip1/jip2* knockout mice did not show a defect in Synapsin 1 distribution [137]. Finally, KHC associates with the AZ protein Liprin-α, as shown by GST-pulldowns, and in *liprin-α* mutants, clear cored vesicles accumulate in axons as well as the SV proteins Synaptotagmin and Synaptobrevin, phenotcopying the *khc* mutant phenotype, possibly suggesting a direct role of Liprin-α during PV transport [105]. Interestingly, KIF1A (kinesin-3) in vertebrates was shown to interact with Liprin-α [92], suggesting that, possibly, both kinesin-1 and -3 can transport Liprin-α.

Of note, transport of neuropeptide-filled DCVs marked by ANF–GFP, a fusion construct of the rat atrial natriuretic peptide and GFP behaving as an endogenous neuropeptide [138], is abolished in *unc104−/−* mutants, and synaptic terminals are devoid of ANF-positive DCVs, which accumulate ectopically in the soma [103,126]. Also, KIF1A (kinesin-1) in mouse [139] and KHC in *Drosophila* [140] are required for DCV transport, connecting to the idea that parts of the molecular DCV biogenesis and transport machinery are used during PV assembly and transport. In summary, despite immense efforts and manifold experiments investigating the kinesin-mediated anterograde PV transport, it is still debated how to interpret and account for the different phenotypes of kinesin-1 (axon swelling, axonal aggregation of SV and AZ proteins) and kinesin-3 mutants (no axon swellings, accumulation of SV and AZ proteins in the cell soma) [1,2,82,118,124]. Future comparative studies using a unified set of PV markers and cargos including both SV and AZ proteins across species might aid in establishing a comprehensive picture of axonal PV transport.

Synapse growth and maintenance are dependent on the contingent of locally available presynaptic protein. Decreased or impeded axonal transport feeding presynaptic material into the synaptic terminal, e.g., in absence of the anterograde microtubule motor kinesin-3 (KIF1A/Unc104) [34,40,103,104], kinesin-1 (KHC/Unc116) [99], or the kinesin adapter Arl8 [35,70], causes severe defects in neurotransmission due to impaired synaptogenesis leading to a reduction of synapse numbers and size. Consequently, several neurodegenerative and neurological diseases are caused by defective anterograde presynaptic protein transport and delivery. Mutations of the anterogradely transporting MT motor kinesin cause amyotrophic lateral sclerosis (KIF5A [141]), microcephaly (KIF5C and KIF2A [142]), peripheral neuropathy “Charcot Marie Tooth Disease Type 2A” (KIF1B [91]), and encephalopathy and brain atrophy (KIF1A [143,144]). Furthermore, mutations of kinesin-binding or -modulating proteins account for the Goldberg–Shprintzen syndrome, causing microcephaly and mental retardation (kinesin-3 inhibitor KBP, [145]) and pontocerebellar hypoplasia (TBC1D23, regulated by Arl8, [146]). Both Alzheimer’s disease (and other forms of dementia) and Parkinson’s disease are at least in part associated with axonal transport defects [147,148].

## 6. The Arl8/BORC Complex and Presynaptic Precursor Vesicle Transport

Cargo specificity of a kinesin can be determined by adaptor or linker proteins associating a specific cargo to a specific kinesin. This paragraph highlights and attempts to summarise an intensely characterised adaptor complex implied in PV transport studied in different species: the Arl8/BORC complex.

Arl8 is a conserved Arf-like small GTPase (ADP ribosylation factors = Arfs), and Arfs form a subfamily of small GTPases mainly implied in the regulation of membrane trafficking [149]. Arl8 is a highly conserved protein from human to plants, with two paralogs (Arl8A and Arl8B) in vertebrates and a single Arl8 (or Gie for GTPase indispensable for equal segregation of chromosomes) in *C. elegans* and *Drosophila* [150]. Arl8 was shown in *C. elegans* to regulate SV-protein-carrying PV trafficking by activation of Kinesin-3 (KIF1A/Unc104) by unlocking the kinesin autoinhibition [115]. Elegant force measurement studies in *C. elegans* revealed that Rab3 transporting PVs can bind more Kinesin-3 (KIF1A/Unc104) motor proteins in presence of Arl8 than in its absence [151]. In *arl8−/−* mutant worms, the SV protein Rab3, the AZ proteins RIM/Unc10, Liprin-α/Syd-2, and a calcium channel subunit accumulated in proximal and were lost from distal axons, a typical transport defect phenotype [42,70], although in *unc104−/−* mutants, Liprin-α/Syd-2 and partially also RIM/Unc10 axonal distribution was not altered [27]. Of note, the AZ protein, ELKS-1, does not require Arl8-mediated transport, as its axonal distribution was not altered in *arl8−/−* mutant worms [27]. In *Drosophila*, Arl8 was detected by axonal life imaging on AZ- and SV-protein-transporting PVs, and in *arl8−/−* mutants, both SV and AZ proteins accumulated in the neuronal cell body and were absent from the synaptic terminal, while axonal trafficking was impeded [35]. In addition, analysis of rat hippocampal neurons showed a partial co-transport of Arl8 and the SV protein Synaptogyrin, but only for 10% of all Synaptogyrin-positive vesicles [152].

Originally, Arl8 was described as a lysosomal resident and adaptor protein to control lysosome positioning and fusion events both in vertebrates and invertebrates [129,150,153,154,155], raising the possibility that a similar transport mechanism is used for both PVs and lysosomes. Interestingly, a recent study suggested a lysosome-like identity of PVs, as they co-trafficked with lysosomal markers [35]. Live imaging analysis revealed that the lysosomal proteins Lamp1 and Spinster co-trafficked with BRP or Synaptotagmin in *Drosophila*, and in mouse hippocampal neurons, Lamp1 was transported together with Bassoon and VGlut (85% of VGlut-positive anterogradely trafficking vesicles are Lamp1-positive and 40% of Bassoon-positive vesicles) [35]. A later in vivo study of axonal trafficking analysis in rat or mouse hippocampal neurons comparing all anterogradely trafficking vesicles showed that 5–25% were co-trafficking events of the SV markers Synaptogyrin or Synaptophysin and Lamp1, while the majority of SV-protein-positive vesicles did not contain a Lamp1 [152]. Future comparative and quantitative studies will have to further characterise the cargo and membrane identity of the axonal PVs.

In vertebrates, Arl8B is recruited to the lysosomal membrane by the BORC complex (biogenesis of the lysosome-related organelle complex 1 (BLOC-1)-related complex) [156]. The BORC complex is composed of eight subunits, some unique to the BORC complex, e.g., Myrlysin, Diaskedin, and Lyspersin, and others shared with the BLOC-1 complex, e.g., BLOS1/2. The BLOC-1 complex is implied in the formation of lysosome-related organelles, while the BORC complex was shown to control lysosomal positioning [154,156], lysosomal size, and reformation dependent on the nutrition status via PIKfyve [157] and autophagosome-lysosome fusion [158]. In HEK cells, only the C-terminal Lyspersin domain was sufficient to recruit Arl8B to the lysosomal membrane and to control peripheral lysosomal positioning [154]. Furthermore, Arl8B in vertebrates and Arl8 in *Drosophila* were shown to interact with the adaptor protein SKIP (SifA and kinesin-interacting protein) to hook lysosomes onto KIF5 (kinesin-1 family), thus allowing for an MT plus-end-directed outward movement of lysosomes [150,159,160,161]. Arl8 recruits SKIP by binding to its PHdomain to the lysosomal membrane, where it relieves SKIP autoinhibition to allow for Kinesin-1 binding and lysosomal outward trafficking [162]. Arl8A or B overexpression in vertebrate cells caused an accumulation of lysosomes at the cell periphery due to increased lysosome mobility [159,161]. In vertebrates, the BORC complex/Arl8B/SKIP lysosomal adaptor ensemble is a general regulator of lysosomal motility, as depletion of the single components, e.g., Myrlysin or Lyspersin, affected polarised lysosomal movement in axons [163]. Amounts of lysosomes were decreased upon knockdown of the single components, while overexpression of the KIF5A (kinesin-1 family) and the Kinesin light chain (KLC1) together with SKIP caused the axonal accumulation of lysosomes. Of note, further experiments revealed that the Arl8/BORC can interact with both kinesin-1 and kinesin-3 family members for lysosomal positioning in distinct cellular localisations, dependent on the type of MT present [164]. While KIF5B (kinesin-1) moves lysosomes around the nucleus along the acetylated MT tracks, KIF1A and KIF1B (kinesin-3) are responsible for peripheral lysosome movements on tyrosinated MTs [164]. Also in *Drosophila*, the BORC complex subunits (Blos1/2, Snapin) were shown to be required for Arl8 localisation and activity to regulate lysosomal positioning, autophagosome–lysosome fusion, and endosome–lysosome fusion [155]. As Arl8 is required for PV transport, and PV shares a lysosome-like membrane identity, the question was raised of whether the BORC complex is required during Arl8/Kinesin-3 (Unc104)-dependent PV transport.

In *C. elegans*, depletion of specific components of the BORC complex, e.g., BLOS-1/2 and Myrlysin, phenocopied the Kinesin-3 (KIF1A/Unc104) mutant phenotype, and SVs accumulated ectopically in the proximal axon [165]. However, not all BORC complex components (Diaskedin, KXD1) were required in this process, implying a subunit-specific requirement of the BORC complex. Here, the BORC complex seems to act upstream of and in the same pathway as Arl8 and KIF1A/Unc104, as an increase of Arl8 or KIF1A/Unc104 activity by expression of constitutively active alleles rescues the BORC-mutant phenotype [165]. In vivo experiments have shown that Arl8 is recruited to PVs by Myrlysin and, in vitro, Myrlysin activates Arl8 by promoting the GDP to GTP exchange [165]. In contrast, in vertebrates, upon knockout of the BORC subunit Myrlysin from mouse hippocampal neurons, the SV proteins SV2 and Synaptogyrin were still present in the axon and reached the presynapse, while only Lamp1, as a marker for lysosomes, was absent from the axons [152]. Consistently, in vivo analysis revealed axonal trafficking defects for Lamp1, but not Synaptogyrin, suggesting that in the vertebrate system the BORC complex is not required for PV, but specifically for lysosomal transport, arguing for a species-dependent requirement for the PV/kinesin adapter complex.

## 7. Conclusions and Outlook

Presynaptic precursor vesicles raised scientific interest already in the 1980s and a wealth of experimental data has been collected to date, fusing into a widely accepted model derived from several independent experimental approaches ranging from live imaging over ultrastructural electron microscopic to immunofluorescence labelling analysis across different species. It is likely that PVs traffic at least to some extent as multivesicular complexes or transport packets composed of unitary vesicles with different morphologies, including 40–60 nm sized clear or slightly dense cored vesicles and 80 nm sized dense cored vesicles (Figure 2C). Additionally, a minority of PVs traffic solitarily; these are presumably 80 nm sized vesicles with the typical dense core (Figure 2C). Several lines of evidence suggest that AZ proteins and presynaptic membrane proteins are predominantly charged on the large 80 nm sized vesicles, but peripheral membrane proteins of SVs like Rab3 were also detected on this vesicle type. A majority of SV proteins, however, seems to be transported by the smaller clear cored vesicles. Nonetheless, the PV field would highly profit from a broad and consistent ultrastructural analysis of PVs in combination with an immunodetection of a full set of presynaptic proteins including AZ, SV, and presynaptic membrane proteins.

Regarding the mechanisms and molecular pathways governing PV assembly, early and recent findings synergise in a common model, suggesting that PV biogenesis occurs at the exit sites of the Golgi, the *trans*-Golgi. Small GTPases, including Rab2, likely control early steps of PV assembly, possibly recruiting effector proteins to the nascent PV membranes and thus initialising vesicle budding from the Golgi, and possibly also directing cargo load and sorting. Interestingly, mature PV membranes seem to share, to some extent, a lysosome-like identity and require the lysosomal kinesin adaptor Arl8 for PVs export from the neuronal soma. This raises the interesting possibility that additional fusion events with components of the endo-lysosomal system could contribute to the PV maturation. Finally, as Rab2 plays a pivotal role in dense core vesicle maturation at the *trans*-Golgi, molecular mechanisms and molecules of the DCV assembly pathway might contribute to PV biogenesis, a promising direction for future investigations (Figure 3).

Anterograde PV transport along the axon to the consuming synapse is performed by kinesins. Live imaging experiments using fluorescently tagged presynaptic proteins in combination with genetic mutations of the different kinesins raised a substantial amount of experimental data deciphering which kinesin and kinesin adaptors are responsible for PV transport (Table 2). Clearly, kinesin-3 family members seem to play a major role in PV transport, although in vertebrates, KIF1A was mainly shown to transport SV proteins, and in invertebrates, Unc104 was shown to be required for both AZ and SV proteins. Additionally, adaptor proteins for kinesin-1 family members were shown to be required for AZ protein transport in both vertebrates and invertebrates; however, in the absence of KHC (kinesin-1 family), microtubules themselves are distorted and this could be the primary cause for observed PV trafficking defects. Future, comparative studies in vertebrate and invertebrate species mutating kinesins and kinesin adaptors monitoring a full set of presynaptic proteins would certainly unify the divergent punctual observations.

## Figures and Tables

**Table 1:** Presynaptic proteins according to their localisation, function, and membrane binding or association properties. This table is not a complete list of all presynaptic proteins but highlights the proteins mentioned as PV cargos in this review.

	Presynaptic Protein
**Cytosolic proteins**	RIM-BP (Rab3-interacting molecule binding protein)—AZ scaffold protein.
	Piccolo—AZ scaffold protein (vertebrates).
	Bassoon—AZ scaffold protein (vertebrates).
	ELKS/CAST (glutamic acid (E), lysine (K), leucine (L) and serine (S)-rich protein = ELKS; cytomatrix at the active zone (CAZ)-associated structural protein = CAST) protein family (homologue Bruchpilot (BRP) in *Drosophila*)—AZ scaffold protein.
	Liprin-α/Syd-2 (LAR-interacting protein = Liprin; synapse defective 2 = Syd-2)—AZ scaffold protein.
	RIM (Rab3-interacting molecule)—release factor.
	(M)Unc13 (uncoordinated movement 13)—release factor.
	(M)Unc18 (uncoordinated movement 18)—binds Synaptobrevin.
	SNAP-25, Syntaxin (SNARE complex).
**Integral membrane proteins (presynaptic membrane)**	Voltage-gated Ca^2+^ channels (VGCC or Cavs).
	SNAP-25 (synaptosomal-associated protein, 25 kDa) (SNARE complex).
	Syntaxin (SNARE complex).
	Neurexin.
	N-Cadherin.
**Integral membrane proteins (SV)**	Synaptobrevin/Vamp (SNARE complex).
	Synaptotagmin/p65.
	VGlut (vesicular glutamate transport protein).
	Synaptophysin.
	Synaptogyrin.
	SV2 (synaptic vesicle protein 2).
**Associated membrane proteins (SV)**	Rab3 (Ras-associated binding 3).
	Synapsin.
	CSP (cysteine string protein).

**Table 2:** Summary of experimental evidence describing the presynaptic protein cargos transported by or interacting with kinesin-1 and kinesin-3 in vertebrates and invertebrates. Transported proteins are named using the following colour code: presynaptic membrane proteins (magenta), presynaptic active zone proteins (blue), synaptic vesicle proteins (green), voltage-gated calcium channels (orange), all proteins bold. If investigated, but not detected as a cargo, proteins are marked in italics. This table also contains presynaptic cargos linked to a kinesin via an adaptor. Additionally, kinesin mutant or gain of function phenotypes are mentioned briefly, as well as relevant experimental data questioning a transport function.

	kinesin-1 KIF5 Vertebrates, KHC *Drosophila*, Unc116 *C. elegans*	kinesin-3 KIF1 vertebrates, Imac or Unc104 *Drosophila*, Unc104 *C. elegans*
**Vertebrates**	KIF5B: **Syntaxin 1a** trafficking depends on the kinesin-1 adaptor Sytabulin (Su et al., 2004 [86]).	KIF1A: **Synaptotagmin, Synaptophysin, Rab3A** (*not **SV2, Syntaxin 1a, SNAP-25***) (Okada et al. 1995 [87]).
	KIF5B: **Bassoon** trafficking depends on the kinesin-1 adaptor Sytabulin (Cai et al., 2007 [88]).	KIF1A: **Synaptotagmin, SV2** (Yonekawa et al., 1998 [34]).
	KIF5B: interacts with **SNAP-25** (yeast-2-hybrid) (Diefenbach et al., 2002 [89], Morton et al. 2010 [90]).	KIF1B: **Synaptotagmin, Synaptophysin, Rab3A** (*not **Syntaxin 1a, SNAP-25***) (Zhao et al. 2001 [91]).
	KIF5A/B: associated with SVs (mass spectrometry) (Takamori et al., 2006 [25]).	KIF1A: direct interaction with **Liprin-α** and in a complex with **RIM** (Shin et al., 2003 [92]).
	KIF5C: **Syntaxin 1a** (Chua et al. 2012 [93]) and **Bassoon**, **Piccolo**, **(M)Unc13** (Butkevich et al., 2016 [94]) via FEZ adaptor.	KIF1A: **Synaptophysin, Synaptobrevin/Vamp, SV2** (Sgro et al. 2013 [95]).
		KIF1A: **Synaptophysin** (Guedes-Dias et al. 2019 [96]).
**In-** **vertebrates**	KHC: *khc* −/− mutants with reduced number of synapses, paralyzed, axon swellings containing **Synaptotagmin**, **CSP**, **Syntaxin** (Saxton et al., 1991 [97]; Gho et al., 1992 [98], Hurd et al.,1996 [99]).	Unc104: less SVs at presynapse and accumulation in the cell body in *unc104* −/− mutants, *C. elegans* (Hall and Hedgecock 1991 [100]).
	KHC: **Synaptotagmin** and **CSP** via FEZ adaptor (Gindhart et al., 2003 [101], Toda et al., 2008 [102], Chua et al., 2012 [93]).	Unc104/Imac: less SVs at presynapse and accumulation in the cell body in *unc104* −/− mutants, *Drosophila* (embryo, larva) (Pack-Chung 2007 [103], Kern et al. 2013 [104]).
	KHC: associates with **Liprin-α** (Miller et al., 2005 [105]).	Unc104/Imac: **Synaptotagmin**, **VGlut**, **BRP** (Pack-Chung 2007 [103], Kern et al. 2013 [104], Zhang et al. 2016 [106]).
	KHC: **Synaptotagmin**, **Synaptobrevin** (Horiuchi et al., 2005 [107] and 2007 [108]) and **BRP**, **RIM-BP** (Siebert et al., 2015 [39]) trafficking regulated by APLIP-1 adaptor.	Unc104: **Synaptotagmin**, PH domain-mediated binding of Unc104 to the (PI(4,5)P2) in PV membranes is required for PV trafficking (Klopfenstein et al., 2004 [109]).
	KHC: KHC mutant impeding MT sliding function without axon swelling and ***Synaptotagmin, CSP*** trafficking is not disrupted (Winding et al., 2016 [110]).	Unc104/Imac: **Synaptotagmin**, **CSP** (Barkus et al. 2008 [104]).
	KHC/Unc116: ***ELKS-1** and **Liprin-α/Syd2*** traffic independent of Unc116 (Lipton et al., 2018 [27]).	Unc104 and KIF1B: **Rab3** transport dependent on DENN/MADD (Rab3-GEF) (Niwa et al. 2008 [111]).
		Unc104: **Synaptobrevin** transport dependent on Liprin-α/Syd-2 (Wagner et al., 2009 [112]).
		Rab3, **Synaptobrevin**, **Synaptogyrin**, **Liprin-α**, **RIM/Unc10**, **Ca**^2+^ channel transport dependent on Arl8 (Unc104 adaptor/activator) (Klassen et al., 2010 [70], Wu et al., 2013 [42]).
		Unc104: **Synaptogyrin**, *unc104* −/− mutants no anterograde SV protein trafficking (Maeder et al., 2014 [40]).
		Unc104: **Rab3** transport decreased in *unc104* −/− mutants and increased in gain-of-function mutations (Zheng et al., 2014 [113]).
		Unc104: **Liprin-α** and VGCC **Cacophony (Cac)** (Zhang et al. 2016 [106]).
		BRP, **Sytnaptotagmin**, **VGlut** transport dependent on Arl8 (Unc104 adaptor/activator) (Vukoja et al., 2018 [35]).
		ELKS-1 did not require Unc104 or Arl8 for transport, RIM/Unc10 partially (Lipton et al., 2018 [27]).
		Unc104: **Neurexin** (NRX-1) (Oliver et al. 2022 [114]).
		Unc104 (over-activation): SV number increases (Niwa et al. 2016 [115]).
		Unc104 (hyperactivation): causes accumulation of **Synaptobrevin** in the neuronal tip (Chiba et al., 2019 [116]).
		Wallenda (Wnd)/DLK MAP kinase axonal damage signalling pathway activation upon Unc104 depletion mediates synaptic defects, possibly independently of Unc104 transport function (Li et al., 2017 [117]).
	*Kinesin-1(khc)/kinesin-3 (unc104)* double mutants: ELKS-1 still traffics along the axon (Lipton et al., 2018 [27]).	

## Data Availability

No new data were created.
